# Study of Sustainability and Health-Related Living Environment Factors Under the Background of Rural Revitalization in the Towns of Guzhen County

**DOI:** 10.1155/sci5/2154665

**Published:** 2024-12-19

**Authors:** Lei Dang, Haoyu Gu, Weishen Wang, Xiaoling Cheng, Soobong Kim

**Affiliations:** ^1^Department of Architecture, Graduate School, Keimyung University, Daegu 42601, Republic of Korea; ^2^Department of Landscape Architecture, Henan Agricultural University, Zhengzhou 450002, China; ^3^Department of Art Artist Design, Art School, Anhui Jianzhu University, Hefei 230009, China

**Keywords:** Anhui, chi-squared test, healthy living environment, human settlement, sustainability, rural revitalization

## Abstract

It is crucial to prioritize research and development as part of rural revitalization efforts to promote balanced economic growth, preserve cultural heritage, and reduce urban migration. This ensures sustainability and encourages equal access to opportunities, resources, and a healthy environment for people in both rural and urban areas. For the first time, this study explores the interplay between sustainability (Factor A) and health-related environment (Factor B) under the background of rural revitalization in the eight towns (Chengguan, Haocheng, Liacheng, Renqiao, Hugou, Xinmaqiao, Liuji, and Wangzhuang) located in Guzhen County, Bengbu City, China. The towns of Guzhen County were not studied under these two factors before. This study evaluates the sustainability and health-related environmental needs by gathering data from 100 residents in eight towns, totaling 800 residents from the targeted study area. Sustainability highlights town-specific priorities; however, health-related needs (Factor B) emphasize the mutual needs of all residents. Similar results in different towns highlight the critical significance of health-related environment. Statistical analysis reveals a significant correlation between sustainability and health-related environmental factors. This stimulates further exploration of nuanced dynamics in rural revitalization. A higher agreement percentage (80%–100%) on health-related aspects underscores the importance of prioritizing policies to overcome these issues in the study area.

## 1. Introduction

In 2017, a rural revitalization strategy was introduced in China [1–3]. By proposing and executing the rural revitalization strategy, China addresses the pressing need to bridge the urban–rural development gap, tackles the challenges inherent in the market system, and fosters modernized agriculture. The rural revitalization plan is pivotal in propelling the high-quality evolution of agriculture and rural regions [4–6]. Hence, meticulous planning emerges as the fundamental prerequisite for the pursuit of rural reinvigoration. There is a growing focus on environmental and health issues in current national and international research, particularly in reducing significant causes of the world's health problems.

**Figure 1:**
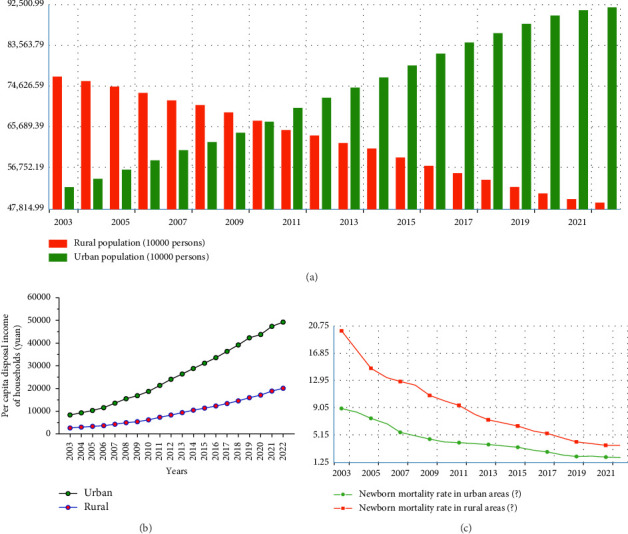
(a) Changes in urban and rural population distributions, (b) per capita disposal income, and (c) newborn mortality rate in rural and urban areas of China in the last 20 years. Source: China Statistical Yearbook 2003–2022.

China has experienced a significant phase of rapid urbanization in the 21st century, resulting in an apparent divide between urban and rural development [7–9]. Due to this gap, cities have better access to healthcare, education, and employment opportunities than rural areas. Cities also have access to better development platforms. As a result, a sizeable portion of the population has moved to urban areas, particularly larger ones, which has led to a loss of talent in less developed rural areas and smaller towns. This talent migration has slowed development in essential fields such as technological innovation, talent cultivation, and general development, leaving these fields undynamic. At the same time, major cities' rapid growth has been fueled by the influx of talent, resulting in an urban expansion into suburban areas. The vibrancy of urban centers has been diminished by several issues brought on by this rapid urbanization process, such as traffic congestion, ecological degradation, and environmental pollution [10–16]. It is essential to address these issues to achieve a sustainable and balanced development strategy that benefits both urban and rural areas and promotes holistic growth and prosperity. Many places now have nostalgic feelings associated with experiences of clean rivers, clear skies, and abundant vegetation [10, 17]. Urban planners constantly wonder whether urban development will inevitably result in environmental degradation because urban and rural areas represent two very different ways of life [18]. The scientific perspective examines the complex relationships between urbanization and the environment, considering any consequences and the effects of urban expansion on natural ecosystems.  Figure 1 shows the changes in urban and rural population distributions (Figure 1(a)), per capita disposal income (Figure 1(b)), and newborn mortality rate (Figure 1(c)) in rural and urban areas of China in the last 20 years. The results illuminate the imperative to enhance the quality of life in rural areas of China, including human settlement and healthy living environments and facilities.

Rural areas are integrated entities with natural, social, and economic characteristics, serving multiple functions such as production, livelihood, ecology, and culture [19, 20]. They interact and coexist with urban areas, forming the main spaces for human activities. Overall, rural areas of countries face a generally lower quality of living conditions. The main issue stems from the increasing desire for quality of life among the residents, which is uneven and inadequate development in rural regions [21]. It is suggested that improving the living environment is a crucial aspect of rural revitalization [22–24]. These health-related environmental challenges are encountered worldwide, and countries are addressing these issues through various strategies [25–27]. A comprehensive study by Agboola et al. reveals a strong positive link between the use of market squares and the residents' sense of community and quality of life [28]. The framework can be efficiently utilized by landscape designers, architects, and related professionals [29–31].

The study of healthy living environments is an active field of research worldwide, dealing with various challenges. A survey by Hafeez et al. [32] investigates Pakistan's potential health trends and disease burden from the last 3 decades (1990–2019), showing improvements in life expectancy and reductions in some health disparities. It identifies the main reasons for mortalities, such as malnutrition and air pollution. The research indicates the need for reasonable health investments and policy interventions to address such critical issues to improve health outcomes [32]. This study also shows the importance of exploring such areas in each country. Human immunodeficiency virus (HIV) is known and reported as a public health significance in Latin America. A study that revealed significant approximations of HIV mortality discovered noteworthy spatial variation and deviating local trends with the time factor and age factor [33].

Furthermore, the healthcare system faces noncommunicable diseases (NCDs) as its new challenge in Iran, necessitating a coordinated multisectoral approach [34]. Recent studies have emphasized the impact of the COVID-19 pandemic on numerous factors in society, highlighting the benefits of social media activities and the use of the internet, which played vital roles [35–39]. Several studies demonstrate that technology has enabled information distribution, supported remote work and education [35, 40], and fostered social connectedness and awareness during the pandemic. Such technologies should be incorporated in small towns and cities to avoid spreading diseases and spread awareness [40–43].

The rural living environment and residents' physical and mental health are essential for the country's overall progress [44]. Promoting a human settlement (sustainability) and a healthy rural living environment should be an integral part of implementing the rural revitalization strategy. However, the current environmental conditions in rural areas in China are still not optimistic, with considerable differences across different regions of the province [45]. In addition, some interesting phenomena have been observed, where after the construction of beautiful rural areas, while there have been improvements in the environmental appearance, there is low community involvement in public service facilities and severe resource underutilization [46, 47]. The current status of the rural living environment after renovation does not match the expectations, and there is a misalignment between the supply and demand of the residents. Such problems can be addressed from the perspective of social innovation design. The area where everyone participates in design calls for designs that address and imagine new ways to solve social problems and create a new future. Therefore, designing a healthy rural living environment is more than just environmental renovation because it involves not only the design of the physical aspect but also the consideration of the users, consumers, and their interests and needs [48]. Involving local residents in the process of developing a strategy for rural revitalization is essential. Guzhen County, located in Bengbu City, Anhui Province, China, is significant on the local level primarily as an agricultural hub. It benefits the community, culture, and economy of the area. However, rural areas of Guzhen County are still not fully developed, necessitating development efforts.

This study is rooted in the environmental design discipline, combining fields such as sustainability (human settlement) and the healthy living environment of Guzhen County, for the first time. We have examined the needs and interests of eight towns (Chengguan, Haocheng, Liacheng, Renqiao, Hugou, Xinmaqiao, Liuji, and Wangzhuang) located in the Guzhen County regarding human habitation and healthy living environment. This study aims to give insight into improving and redesigning rural living areas that meet the living needs of rural residents in Guzhen County. The study will develop specific design and optimization strategies and conduct postuse evaluation and performance assessment studies by employing statistical tests, as well as quantitative, qualitative, and case study methodologies [49].

## 2. Problems Faced in Guzhen County

### 2.1. Unbalanced Development of Urban and Rural Areas

The major obstacle to effectively executing the rural revitalization strategy is the current uneven and inadequate development between China's urban and rural areas, resulting in various issues (Figures 1(a), 1(b), and 1(c)). Notably, according to data from the National Bureau of Statistics, the per capita income ratio between urban and rural residents in 2022 is 2.56; it shows a declining trend over time but is still present at a significant level (Figure 1(b)) [50]. This significant income disparity highlights the wide income gap among residents. Several towns and villages working on rural revitalization face significant historical deficits in infrastructure development, including water, electricity, gas, roads, and network systems [51]. Rural areas also progress more slowly than urban areas do. The problem is worsened by inadequate investment issues and undesirable construction [50]. Notably, challenges vary in rural sewage treatment and waste management facilities, emphasizing the apparent deficiencies.

Last but not the least, rural areas have difficulty providing the infrastructure and essential public services their residents desire for an improved quality of life. Basic public services such as healthcare, education, culture, sports, health, and social security are underdeveloped in rural areas when compared to urban areas. Rural settings' notably poor basic education standards and medical and health services are particularly concerning.

### 2.2. Examining the Spectrum of Issues in Rural Development

Regarding human settlement and fostering a healthy living environment, rural areas, including places such as the towns of Guzhen County, frequently face various difficulties. Circumstances such as inadequate infrastructure, resources, and service access may cause these difficulties. Many issues affecting human settlement (sustainability) and developing a healthy living environment affect rural areas such as Guzhen County, since there are few employment options, incomes are lower, and economic growth is limited. Although agriculture still forms the backbone of rural economies, using outdated farming techniques, having limited access to modern equipment, and the vagaries of unpredictable weather patterns all together present significant challenges. Poor living conditions, inadequate sanitation systems, and restricted access to necessary social amenities threaten residents' overall welfare. Deficient communication grids and transportation infrastructure make the developmental trajectory even more challenging, increasing societal isolation. Farming methods lead to ecological problems such as deforestation, contaminated water, and soil degradation [52–54]. These worries contribute to the aging of rural populations and the decline in vitality in these areas as younger demographics migrate to urban areas in search of better opportunities [55]. Integrating local, regional, and national strategies is required for a comprehensive approach to address these complex problems effectively. This includes enhancing the basic infrastructure, offering accessible services, and promoting sustainable development principles.

### 2.3. Gaps in Knowledge: The Need for Empirical Research in Rural Development

Residents of China now prioritize fitness, environmental protection, leisure, and concerns about the ecological environment [56]. With the development of modern science and technology, humanity has entered the information age [57]. People have gradually understood that protecting the environment is crucial for ensuring human survival, especially with the formation and development of environmental science and ecology. More than just physical environmental changes are involved in designing a healthy rural living environment. Professionals in research and design must participate, comprehend what is currently going on, and learn from successful examples and experiences. To diagnose the rural living environment for rural revitalization, we must collect data from various sources and conduct focused research from multiple perspectives. We can address the health needs of vulnerable rural communities and aid in rural revitalization through empirical research. This entails giving advice and promoting advancements in strategy-making, decision-making, and the creation of growing green infrastructure in rural areas. Every researcher and professional involved in environmental design needs to carefully consider these important questions. The medical and health industries in rural areas were the main subjects of health research in the past. However, it is clear from a review of the literature that scientists working in fields such as environmental design and landscape architecture have contributed unique viewpoints and focused research trends [58, 59]. However, there is a lack of empirical research in this field, both within the Anhui Province and nationwide. Moreover, research focused on the rural living environment from the perspective of public health, particularly concerning the assessment of physical, psychological, and social health aspects of rural residents, has significant room for exploration. In this work, a combination of questionnaires and interviews is developed to delve into the experiences and viewpoints of the local population from the eight towns of Guzhen County for the human settlement factor and healthy living environment. These methodologies enable a thorough investigation of the prevalence, diagnosis, development, and overall management of human settlement and healthy living environments in this community under rural revitalization, as well as the direct effects of inadequate infrastructure, restricted access to healthcare services, and other issues.

## 3. Data and Methodology

### 3.1. Study Area

**Figure 2:**
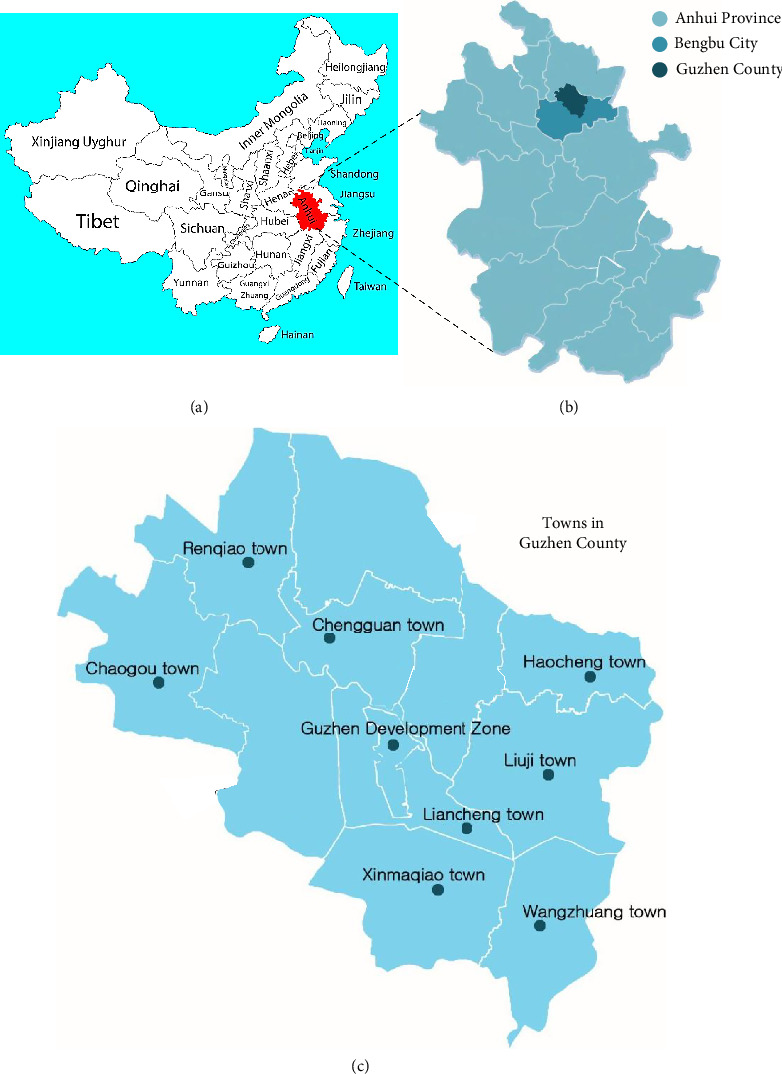
The study area map: (a) Anhui Province, (b) Bengbu city in the Anhui Province, including Guzhen County, and (c) the targeted eight towns in Guzhen County.

Bengbu City is situated within the Huaihe River Basin, located in the northern region of Anhui Province, as shown in Figure 2(a), and encompasses three counties: Wuhe, Huaiyuan, and Guzhen. The total land area spans 5952 km^2^, bordered by Mingguang City to the east and Mengcheng County to the west. Within the rural expanse, Longkang Farm has garnered accolades as a nationally recognized agricultural standardization demonstration farm, exemplifying a model of eco-village construction [60, 61]. While the city's development trajectory bears significance, the current emphasis lies not solely in planning but in addressing the challenges of rural ecological environmental protection in Bengbu City [62]. Bengbu was a significant city in China that was known for its industries and transportation connections. Concerning the distribution of industrial enterprises, the three counties collectively host 358 above-scale industrial establishments, accounting for 61.4% of the total number, accompanied by progressive growth in industrial output. However, investment in urban environmental infrastructure significantly surpasses that allocated to rural areas, leading to a notable disparity between the two. As a result, it is still critical to address Bengbu City's rural ecological and environmental challenges [63, 64]. Guzhen County, located within Bengbu City (Figure 2(b)), has eight towns, as shown in Figure 2(c). The study centers on these eight towns within Guzhen County: Chengguan, Haocheng, Liacheng, Renqiao, Hugou, Xinmaqiao, Liuji, and Wangzhuang. These towns have been chosen due to their notable importance and pressing improvement needs.

### 3.2. Methods

This study is based on professional backgrounds in environmental design and landscape architecture. It utilizes empirical research on environmental health, landscape perception preferences, and theories related to healthy social life. The study employs sociological and multidimensional research methods such as questionnaires and interviews. It selects typical rural areas in Bengbu City, investigates villagers' living and working environments, and conducts qualitative and quantitative assessments of physical health, psychological well-being, and social interactions. The study also introduces the concept of functional amenities to analyze the relationship between the physical environment characteristics of rural areas and villagers' health behaviors.

The human element is the core of scientific research on the living environment. By studying human settlement patterns, we explore how humans interact with nature and their surrounding environment, forming a comprehensive management system. The five elements that define the living environment are humans, society, nature, architecture, and networks. Rural habitation was assessed by considering two distinct factors, denoted as Factor A (sustainability) and Factor B (health-related living environment). Sustainability encompasses the dimension of human settlement, while heath-related living environment pertains to the quality of the living environment regarding health, as shown in Table 1. The study was conducted in eight towns in Guzhen County. The selected towns are as follows: Chengguan, Haocheng, Liacheng, Renqiao, Hugou, Xinmaqiao, Liuji, and Wangzhuang. The 100 rural residents from each city were surveyed to gather insights on Factors A and B in Guzhen County (800 people). Subsequently, the collected data were analyzed, focusing on the individual needs and concerns of each town in Guzhen County. The obtained results were compared with the disagree, neutral, and agree percentages on the evaluation items in Table 1 for Factors A and B. This comparison aims to discern their respective significance levels, providing valuable insights for understanding the initial steps required for development in this region under rural revitalization.

### 3.3. Statistical Methodology Employing the Chi-Square Independence Test

In analyzing rural revitalization factors, we deal with categorical and qualitative data, specifically about sustainability or human settlement and heath-related living environment. Considering the properties of our dataset, a nonparametric test is the most proper statistical approach. The chi-square test can widely assess the relationships between two categorical independent variables [65]. Several studies have previously employed this technique [43, 65, 66]. We have used the chi-square test of independence to estimate an in-depth dynamic related to rural revitalization. Two critical factors named “sustainability or human settlement” and “health-related living environment” are the primary focus of this work in the eight towns of Guzhen County.

#### 3.3.1. Formulation of Hypotheses

The null hypothesis *H*_0_ suggests that sustainability or human settlement and health-related living environment are significantly related to the considered factors. In this initial study of the towns in Guzhen County, we have chosen to use the chi-square test and estimated frequencies due to their suitability for our current dataset and research scope. In contrast, the alternative hypothesis *H*_*a*_ indicates a significant correlation, which implies that the elements of a health-related living environment influence sustainability. The 0.05 value was established as a threshold for estimating statistical significance between the outcomes.

The test statistic combines data from observed frequencies represented with “O” and expected frequencies indicated with the symbol “*E*” through classifications of categorized variables to execute the chi-square test of independence. The values obtained from each category are summed up to determine the test statistic. The expected frequency (*E*) for each cell was calculated, assuming that sustainability and heath-related living environment do not correlate. The calculation was made by using the equation given as follows:

(1) $$\begin{array}{c} E=\frac{R_{t}\times C_{t}}{G_{t}}, \end{array}$$

where *R*_*t*_ shows the row total and *C*_*t*_ indicates the column total in the contingency table, and *G*_*t*_ refers to the total, which is the total count of observations. When the two factors are independent, equation (1) can be used to estimate the “*E*” for each cell under consideration. First, a contingency table will be produced, and a town-based analysis will be carefully classified based on their opinions to evaluate the sustainability factor (Factor A) and health-related living environment factor. Furthermore, *E* was investigated for each cell under the hypothesis of no correlation between sustainability factor and health-related living environment factor. The calculation follows equation (1)

Chi-square statistic computation: The statistical backbone of the analysis takes shape with the computation of the chi-square statistic indicated as *χ*^2^. The chi-square test statistic *χ*^2^ is calculated by using the formula given as

(2) $$\begin{array}{c} \chi^{2}=\sum \frac{{\left(O_{i}-E_{i}\right)}^{2}}{E_{i}}, \end{array}$$

where *O*_*i*_ is the observed frequency in a category and *E*_*i*_ is the expected frequency in the same category (calculated using the theoretical distribution assumption) which denotes the total of all categories. The degrees of freedom (*d*_*f*_) are calculated as *d*_*f*_ = *k*_*i*_, where *k* is the number of categories. The test statistic can be compared with the critical values, just like the test for independence. A critical decision-making moment occurs when the calculated chi-square statistic is compared with its necessary counterpart. The null hypothesis will likely be rejected if the computed value exceeds the crucial threshold.

## 4. Results and Discussion

### 4.1. Sustainability in Eight Towns of Guzhen County (Factor A)

**Figure 3:**
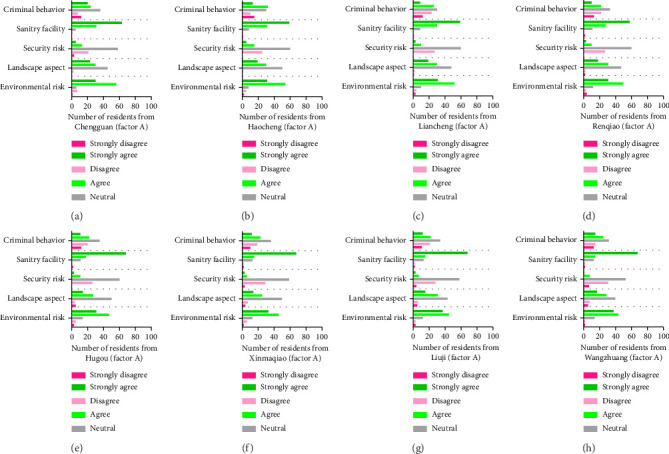
Perceptions of residents from eight towns in Guzhen County regarding sustainability or human settlement (Factor A) were categorized into strongly disagree, disagree, neutral, agree, and strongly agree. The towns are as follows: (a) Chengguan, (b) Haocheng, (c) Liacheng, (d) Renqiao, (e) Hugou, (f) Xinmaqiao, (g) Liuji, and (h) Wangzhuang. The responses indicate a broad spectrum of needs of the residents of the towns when sustainability is considered.

The sustainability of the human settlement factor significantly affects the living conditions, progress, and population of the people residing in rural areas of Guzhen County. Investigating issues about different facets that enhance these communities' living standards is necessary to comprehend this factor (sustainability).  Figure 3 summarizes the results of an extensive analysis that looked into the opinions and interests of the people living in eight distinct towns in Guzhen County. The main goal of this work is to investigate the sustainability factor (Factor A) under rural revitalization. To represent the diversity of opinions, participants from the towns of Guzhen County were asked about the views and options ranging from “strongly disagree” to “strongly agree.” The towns that are being examined are (a) Chengguan, (b) Haocheng, (c) Liacheng, (d) Renqiao, (e) Hugou, (f) Xinmaqiao, (g) Liuji, and (h) Wangzhuang. After thoroughly surveying all participants' responses, a remarkable pattern was observed regarding sustainability or human settlement factors. The results showed a wide range of preferences among the selected residents under study, particularly considering the sustainability factor (Factor A).

Evaluating residents' environmental risk considers potential challenges such as pollution and natural disasters. Analyzing esthetic and visual components such as green areas and the surrounding landscape is necessary for assessing the landscape aspect. Based on the opinions of 100 residents per town (totaling 800 participants from eight cities), Table 2 displays survey results with a particular emphasis on sustainability or human settlement. The respondents were asked to rate their agreement with various statements related to different aspects of a human settlement factor. The responses are categorized into five levels: “strongly disagree,” “disagree,” “neutral,” “agree,” and “strongly agree,” as shown in Figure 3. Here, Table 2 provides the percentage of disagreement, neutrality, and agreement from the eight towns of Guzhen County upon sustainability.

Rural areas play a pivotal role in the socioeconomic landscape, and understanding the dynamics of human settlement within these regions is crucial for practical rural revitalization efforts. Guzhen County, with its eight distinct towns, provides a unique microcosm through which we can analyze the complex interplay of factors shaping human settlements within the rural context.

### 4.2. Assessment of Health-Related Living Environment

**Figure 4:**
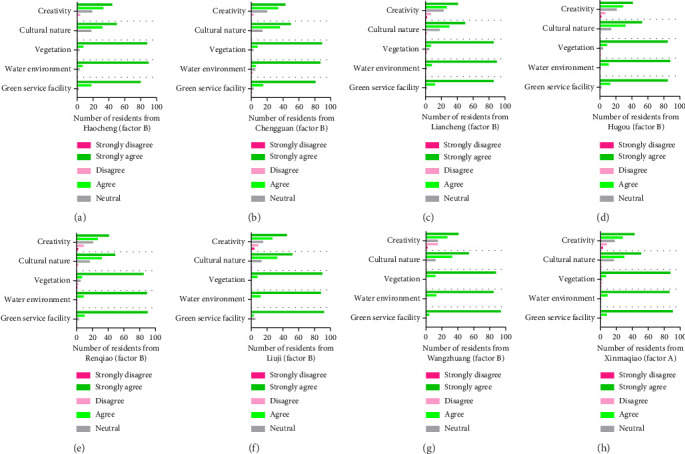
Perceptions of residents across eight towns in Guzhen County about the quality of health-related living environment , categorized along a continuum from “strongly disagree” to “strongly agree.” The towns are identified as follows: (a) Chengguan, (b) Haocheng, (c) Liacheng, (d) Renqiao, (e) Hugou, (f) Xinmaqiao, (g) Liuji, and (h) Wangzhuang. The aggregated responses underscore a high level of agreement about promoting a health-related living environment.

Figure 4 provides the results obtained considering the heath-related living environment. Residents of eight different towns in Guzhen County were surveyed about their responses to the quality of the local living environment, focusing on factors connected to health (heath-related living environment). Participants were asked to rate how strongly they agreed or disagreed with certain statements.  Figure 4(a) indicates the results from Chengguan, (b) Haocheng, (c) Liacheng, (d) Renqiao, (e) Hugou, (f) Xinmaqiao, (g) Liuji, and (h) Wangzhuang. After examining the group responses, a clear pattern becomes apparent. Regarding issues related to establishing a health-centric living environment, there is an apparent convergence of ideas among the people. This consistency in replies reveals a common tendency among the many municipalities in Guzhen County to value characteristics connected to a wholesome living environment.

Table 3 presents the percentage of the evaluation of a questionnaire about the factors influencing a healthy living environment in eight towns of Guzhen County. The respondents were asked to rate their agreement with various statements about a healthy living environment. The responses are categorized into five levels: “strongly disagree,” “disagree,” “neutral,” “agree,” and “strongly agree,” as shown in Figure 4. Furthermore, Table 3 provides the percentage of disagreement, neutrality, and agreement for heath-related living environment from the eight towns of Guzhen County.

The higher agreement percentage of the participants from each town shows its importance and indicates a priority step for rural revitalization in Guzhen County.

### 4.3. Towns Analysis by Incorporating Sustainability and Health-Related Environment

#### 4.3.1. Chengguan

Sustainability (Factor A): Chengguan exhibits a remarkable consensus on several evaluation items. The town displays a substantial 86% agreement on residential environmental risk, reflecting a sense of safety and harmony. Moreover, the prevalent deal (53%) on landscape emphasizes the significance of a balanced and aesthetically pleasing environment. This response makes Chengguan a promising hub for rural revitalization efforts. Health-related environment: The obtained data from Chengguan town indicates an overwhelmingly positive opinion towards its green service facilities (96% agree). This highlights the importance of accessing the outdoor spaces. In addition, Chengguan presents itself as a possible center for creativity and health-focused studies within rural revitalization, with a noteworthy 77% agreement on the value of requiring creativity.

#### 4.3.2. Haocheng

Sustainability (Factor A): Haocheng reveals a notable inclination towards the agreement, particularly evident in its 85% agreement rate concerning residential environmental aspects. A balanced distribution across neutral and agree responses regarding security risk (49% agree and 50% neutral) suggests a stable environment, which could be capitalized on during rural revitalization initiatives. Health-related environment: Haocheng's results reflect its residents' inclination towards fostering a healthy living environment. High agreement percentages for water environment (98%) and cultural nature (82%) highlight a community that values preservation and appreciation of natural and cultural heritage. This forms a solid foundation for initiatives promoting health and local heritage during rural revitalization.

#### 4.3.3. Liacheng

Sustainability (Factor A): Liacheng's responses emphasize the importance of sanitation and hygiene, with a substantial 89% agreement on sanitary facilities. However, there seems to be room for improvement regarding perceptions of criminal behavior (35% agree), indicating an opportunity for targeted interventions. Health-related environment (Factor B): Liacheng's emphasis on the water environment (98% agree) underscores a collective aspiration for a clean and sustainable ecosystem. However, the relatively lower agreement (68%) on fostering creativity opens doors for innovative interventions to infuse creativity into daily rural life and promote holistic well-being.

#### 4.3.4. Renqiao

Sustainability (Factor A): Renqiao's evaluation data exhibit exciting dynamics. While there is a positive inclination towards neutral and agreement with responses on security risk and criminal behavior, the town's relatively lower agreement on residential environmental aspects (32% agree) suggests a need for enhancements in the living environment. Health-related environment (Factor B): Renqiao achieved 100% agreement on the significance of the water environment. With 68% of the participants agreeing that creativity is essential, the town may be able to support artistic endeavors. Human and ecological health can be promoted by maintaining a healthy water ecosystem, a key component of revitalization strategies in the selected study area. Renqiao stands out with unanimous agreement (100%) on the significance of the water environment. While its 68% agreement on creativity suggests the potential for fostering artistic endeavors, the collective emphasis on maintaining a healthy water ecosystem can catalyze ecological and human health within rural revitalization strategies.

#### 4.3.5. Hugou

Sustainability (Factor A): The data gathered from Hugou provides a comprehensive understanding of the water environment, cultural aspects of nature, and green service facilities. Health-related environment (Factor B): Hugou encounters challenges reaching agreement percentages, especially in residential landscapes and security hazards. However, the noteworthy neutrality rate of 50% concerning security risk indicates a potential for change. Through targeted efforts, this town has the potential to shift dynamics towards more favorable perspectives.

#### 4.3.6. Xinmaqiao

Sustainability (Factor A): Responses from Xinmaqiao show a significant divergence. A substantial 39% of the participants rated residential landscape elements as neutral, even though 83% of the respondents strongly agreed with the importance of sanitary facilities. This suggests that these areas must be addressed in agricultural revitalization initiatives. Health-related environment (Factor B): The 99% agreement showed a focus on the water environment, while 71% agreed on encouraging creativity points of participants.

#### 4.3.7. Liuji

Sustainability (Factor A): The Liuji data highlight neutral responses and show balanced opinions across various evaluation items. This is consistent with the ideas of rural revitalization and suggests the possibility of incremental advancements in several areas related to human settlement. Health-related environment (Factor B): The collective emphasis on maintaining a health-related climate is reflected in the agreement percentages across evaluation items. The agreement of 72% on creativity suggests a readiness to explore innovative approaches that can enhance physical and mental health, providing a robust foundation for integrated rural revitalization strategies in Liuji town.

#### 4.3.8. Wangzhuang

Sustainability (Factor A): In Wangzhuang, favorable viewpoints are evident in residential attributes, hygienic facilities, and perspectives on criminal activity. Landscape features and security issues could be addressed to further maximize the town's potential for revitalization. Health-related environment (Factor B): Wangzhuang demonstrates 98% agreement on cultural nature and 68% fostering creativity. These findings highlight the need to preserve cultural heritage and promote a vibrant rural culture. By fostering these elements, the town can utilize its natural advantages for all-encompassing rural revitalization.

### 4.4. Collective and Comparative Insight of Outcomes From the Towns of Guzhen County

**Figure 5:**
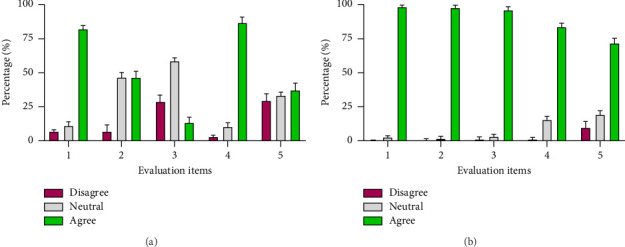
The percentage of disagree, neutral, and agree in (a) human settlement (sustainability) and (b) healthy or health-related living environment. A higher agreement percentage was found in heath-related living environment.

The critical relationship between sustainability (Factor A) and health-related environment (Factor B) is highlighted by the combined and comparative analysis of results from towns within Guzhen County, Bengbu City. This analysis highlights the crucial role of healthy living conditions and human settlement dynamics in forming the overall framework for sustainable rural development. Through a comprehensive understanding of the concerns and aspirations of the local population, policymakers, planners, and stakeholders can develop customized approaches to enhance living conditions while maintaining each town's unique character, heritage of culture, and overall health. Based on each town's specific needs and challenges, sustainability identifies different focal points. Heath-related living environment highlights the significance of creating a healthy living environment as evidenced by the consistently high agreement values among the evaluation items (Figure 5). This comparative analysis pinpoints a shared goal of better health, establishing heath-related living environment as the main force behind gradual transformation and comprehensive landscape revitalization in Guzhen County. The instance about developing a healthy living environment with clean water sources, cultural preservation, and creativity highlights how these towns can be models of integrated wellness and physical, mental, and emotional health revitalization.

### 4.5. Statistical Analysis

When assessing the relationship between two categorical variables, the chi-square test of independence focuses on two critical aspects of rural revitalization: human settlement and heath-related living environment. The goal is to determine whether these factors have a significant relationship. To run the proposed chi-square test, the obtained data were arranged into a contingency table, which gives a tabulation form of the frequencies of mutual occurrences of categories from both the selected study variables. The estimated frequencies' table is essential in preparing the chi-square test for the obtained data. It provides estimated values for each cell that is under consideration without the relationship between sustainability and health-related living environment. Tables 4 and 5 indicate and provide the observed values attained from survey responses of participants (residents) across the eight towns of Guzhen County, Bengbu City, China.

The expected frequency (*E*) for every cell in a contingency table is calculated, assuming independence between the variable categories under study. The chi-square test then compares observed frequencies (O) with these expected frequencies, evaluating whether there is a significant difference from the expected distribution under independence.  Table 6 represents the chi-square test, a reliable technique for examining relationships in categorical data.

#### 4.5.1. Author's Calculations

The chi-square test of independence was used to analyze the correlation between sustainability and heath-related living environment. The results indicate an exciting interdependence between the various opinions of participants of Guzhen County on both factors. The findings of this study create the opportunity for further exploration and understanding of the complex dynamics affecting rural revitalization in the area. It is essential to highlight a higher agreement ratio with consistent opinions across all eight towns regarding heath-related living environment, which is related to the health-related living environment. This emphasizes the significance of heath-related living environment as a focal point of shared values and collective preference. Policymakers should prioritize heath-related living environment, a health-related living environment, due to higher agreement and its relevance [67–70].

### 4.6. Suggestions for Development Steps

Guzhen County shows the critical importance of sustainability and a health-related living environment under a rural revitalization strategy. This means providing aid for the construction of new healthcare facilities as well as the renovation of ones that are already in place [71]. These steps would guarantee that residents would receive quality healthcare since healthcare services would be more accessible and of excellent quality [72]. Guzhen County needs health education, promotion programs, and infrastructure enhancement [73, 74]. Guzhen County can enhance general health outcomes by educating the public about disease prevention, healthy lifestyles, and early intervention [73, 75]. Developing and expanding the county's telehealth infrastructure is necessary to ensure that even the most remote areas can access virtual healthcare consultations [76–80].

Figure 5 holds valuable insights for comparing the overall requirements in Guzhen County. It is noted that a healthy living environment is the top priority of each town in Guzhen County. Moreover, Tables 2 and 3 shed light on the perceptions and priorities of each city concerning various aspects of their living environment. The data presented underline the multifaceted nature of rural revitalization, encompassing the essentials of safety, infrastructure, and services and the aspirations for a more enriching and vibrant quality of life. By analyzing these percentages through the lens of rural revitalization, we can extract meaningful strategies to guide the first crucial steps toward achieving comprehensive community development.

Safety and infrastructure enhancement: The results in Table 2 highlight the community's prioritization of safety and infrastructure. With a significant majority agreeing on the importance of addressing residential environmental risk levels and providing proper sanitary facilities, it is evident that foundational investments in secure living conditions are essential. As a primary development step, authorities should channel resources into comprehensive risk assessments, disaster preparedness plans, and sanitation infrastructure upgrades. This will enhance residents' safety and instill a sense of security crucial for attracting new inhabitants and retaining existing ones.

Esthetic enhancement and quality of life: The responses in Table 3 underscore the significance of enhancing the overall quality of life [64]. The widespread agreement on the importance of green service facilities, water environment, vegetation, cultural nature, and creativity indicates a collective yearning for a more attractive and enriching living environment.

Cultural preservation and economic diversification: The emphasis on cultural nature and creativity in Table 2 presents a unique opportunity for economic diversification and artistic preservation. Integrating local culture into revitalization efforts, such as cultural festivals, heritage preservation, and creative workshops, can enrich the community's identity, drive tourism, and stimulate the local economy. Supporting entrepreneurship and creative industries can further promote job creation and generate additional revenue streams.

Sustainability or human settlement (Factor A): Evaluating human settlement factors reflects the intricate balance between safety, infrastructure, and community cohesion [81]. The responses underline the importance of addressing environmental risks and providing proper sanitary facilities, echoing the fundamental need for secure living conditions [82, 83]. These insights should guide the initial steps in rural revitalization efforts, focusing on risk assessment, disaster preparedness, and infrastructure upgrades to ensure residents' safety and well-being. Disparities in the perceptions of security risk and criminal behavior underscore the significance of community engagement and social inclusion strategies to foster a sense of ownership, reduce crime, and establish a harmonious living environment. To drive rural revitalization, prioritizing these aspects will lay the groundwork for a secure, cohesive, and prosperous community [83, 84].

Health-related living environment (Factor B): Assessing factors contributing to a healthy living environment captures the community's desire for an enriching and active rural lifestyle [85]. The widespread agreement on green service facilities, water environment, vegetation, cultural nature, and creativity highlights the strong preference for an elevated living level. It is well known that environmental risk factors, particularly air and water pollution, significantly impact morbidity and mortality in China [51]. Almost all rural and urban households use coal and biomass fuel for heating and cooking, which causes severe indoor air pollution that significantly increases the disease burden. Since many communities lack access to clean water and proper sanitation, many areas are at a high risk of contracting a disease caused by water [51]. China is rapidly industrializing, causing a corresponding rise in energy consumption and industrial waste production. While this economic development has improved living and health conditions, it has also increased the frequency of environmental disasters and the release of dangerous chemicals. These elements hurt people's well-being. Urban areas in China struggle with some of the worst air quality issues in the world, and industrial water pollution seriously threatens public health. A recent study was reported to enhance the understanding of how social media use impacts academic careers, collaborative learning and awareness, and mental health [86, 87]. These insights provide suggestions for educational strategies and technology policies to improve people's outcomes and health [38, 88].

Furthermore, the rise in greenhouse gas emissions due to energy use is worrisome. Global climate change will likely worsen the nation's environmental health problems, which could have negative consequences [55]. We also have evidence from the collected data that most Guzhen County residents prefer a healthy living environment (Figure 5). Addressing these aspirations necessitates careful urban planning, including green spaces, recreational amenities, and cultural events. The primary goals of rural revitalization are preserving regional culture, encouraging creative industries, and promoting entrepreneurship. The community can be revitalized while maintaining its traditions by embracing these factors, which can turn rural areas into desirable places to visit that combine cultural heritage with economic growth.

The obtained data, derived from questionnaires of 100 residents across eight towns in Guzhen County, may not fully signify the whole population's involvement. The model size limits the generalizability of our findings. Future investigations should involve bigger sample sizes, longitudinal studies, and objective measures to facilitate a more complete dynamic of sustainability and health-related living environments in Guzhen County.

## 5. Conclusions

This study investigates the interplay between the sustainability factor or human settlement (Factor A) and the health-related living environments (Factor B) as fundamental components of rural revitalization planning in Guzhen County, China. The determination draws insights from the 100 individuals (residents) across eight towns of Guzhen County, totaling 800 participants, displaying the impacts of both sustainability and health-related living environments. The results show that the sustainability or settlement factor (Factor A) has town-specific priorities that vary according to the town and individuals. However, the results related to the health-related living environment (Factor B) show uniformity and high agreement percentage across eight towns. The obtained data indicated residents' everyday and urgent needs and suggested the priority steps for policymakers to address in this area under rural revitalization.

Furthermore, significant relations were observed between these two factors (Factor A and Factor B) using the chi-squared test. The results of this study support a long-term and successful implementation of a rural revitalization approach that addresses physical and health-related factors to meet the requirements of Guzhen County. For effective revitalization, strategies must be tailored to the variations in sustainability among towns, highlighting the necessity of targeted, localized interventions. The results of the study indicate that the infrastructure investments that promote a health-related living environment can significantly increase residents' quality of life in Guzhen County. The results also offer practical insights for policymakers in managing the complex rural revitalization efforts in Guzhen County.

### 5.1. Policy Recommendations

Based on the findings of this study, several policy recommendations emerge to support the goal of rural revitalization in Guzhen County, China. The main priority is to invest in the healthcare infrastructure immediately, which requires developing new health-related facilities and renovating/improving existing ones. Implementing health education and promotion activities to educate the residents is also important and helpful. We believe developing community health centers will increase healthcare accessibility and opportunity, mainly through integrating telehealth services to reach local remote areas. Enlarging health insurance coverage for low-income residents and facilitating cooperation between traditional (old) and modern (new) healthcare professionals are also essential. Finally, vigorous inspection and assessment systems should be placed immediately to ensure healthcare facilities' regular advancement and adaptation based on data-driven insights.

### 5.2. Implications

The results of the presented study have important implications for policymakers engaged in the rural development of the study area (towns of Guzhen County). By concentrating on developing a health-related living environment and addressing healthcare facilities as a key and fundamental component, Guzhen County can make considerable progress toward rural revitalization in the future. This study highlights the importance of efforts to build infrastructure and invest in health-related environments and education programs in the towns of Guzhen County. It also indicates the role of community health centers and telehealth in linking accessibility and opportunity gaps. Furthermore, expanding health-related insurance coverage and endorsing collaboration between different healthcare policies presents an extensive approach for improving Guzhen County's health outcomes. The indicated strategies and implications not only solve the urgent healthcare requirements but also play a vital role in the County's long-term sustainability of Guzhen County, aligning with bigger goals of rural revitalization in China.

## Figures and Tables

**Table 1:** The evaluation items for human settlement (Factor A) and heath-related living environment (Factor B).

Evaluation items (questions)	Sustainability (Factor A)	Health-related living environment (Factor B)
1	Environmental risk level	Green service facilities
2	Landscape aspects	Water environment
3	Security risk	Vegetation
4	Sanitary facilities	Cultural nature
5	Criminal behavior	Creativity

**Table 2:** The percentage of disagreement, neutrality, and agreement in each town about human settlement needs, Factor A, in eight towns of Guzhen County.

Evaluation item (sustainability)	Town names (in Guzhen County)	Disagree (%)	Neutral (%)	Agree (%)
Environmental risk level	Chengguan	8	6	86
Landscape aspects		2	45	53
Security risk		24	58	18
Sanitary facilities		1	5	94
Criminal behavior		20	36	44

Environmental risk level	Haocheng	7	8	85
Landscape aspects		1	50	49
Security risk		25	60	20
Sanitary facilities		2	8	90
Criminal behavior		25	30	45

Environmental risk level	Liancheng	7	10	83
Landscape aspects		3	48	49
Security risk		27	60	13
Sanitary facilities		2	9	89
Criminal behavior		35	30	35

Environmental risk level	Renqiao	7	12	81
Landscape aspects		4	47	49
Security risk		27	60	13
Sanitary facilities		3	11	86
Criminal behavior		35	33	32

Environmental risk level	Hugo	8	14	78
Landscape aspects		9	50	41
Security risk		26	60	14
Sanitary facilities		3	11	86
Criminal behavior		32	35	33

Environmental risk level	Xinmaqiao	8	13	79
Landscape aspects		11	50	39
Security risk		32	59	9
Sanitary facilities		4	13	83
Criminal behavior		29	36	35

Environmental risk level	Liuji	6	12	82
Landscape aspects		11	43	46
Security risk		32	58	10
Sanitary facilities		4	13	83
Criminal behavior		32	34	34

Environmental risk level	Wangzhuang	4	14	82
Landscape aspects		14	40	46
Security risk		38	53	9
Sanitary facilities		4	13	83
Criminal behavior		28	32	40

**Table 3:** The percentage of disagreement, neutrality, and agreement in each town about the health-related living environment in eight towns of Guzhen County.

Evaluation item of heath-related living environment	Towns names (in Guzhen County)	Disagree (%)	Neutral (%)	Agree (%)
Green service facilities	Chengguan	1	3	96
Water environment		2	5	93
Vegetation		0	3	97
Cultural nature		0	14	86
Creativity		3	20	77

Green service facilities	Haocheng	0	2	98
Water environment		0	3	97
Vegetation		1	3	96
Cultural nature		0	18	82
Creativity		4	19	77

Green service facilities	Liancheng	0	2	98
Water environment		0	2	98
Vegetation		2	5	93
Cultural nature		2	18	80
Creativity		9	23	68

Green service facilities	Renqiao	0	3	100
Water environment		1	1	98
Vegetation		3	5	92
Cultural nature		2	17	81
Creativity		11	21	68

Green service facilities	Hugo	0	2	98
Water environment		0	1	99
Vegetation		2	4	94
Cultural nature		1	14	85
Creativity		9	21	70

Green service facilities	Xinmaqiao	0	1	99
Water environment		1	1	96
Vegetation		3	2	95
Cultural nature		2	17	81
Creativity		11	18	71

Green service facilities	Liuji	0	5	95
Water environment		0	0	100
Vegetation		1	1	98
Cultural nature		2	13	85
Creativity		13	15	72

Green service facilities	Wangzhuang	1	1	98
Water environment		1	1	98
Vegetation		0	0	100
Cultural nature		1	12	87
Creativity		17	15	68

**Table 4:** Contingency values of sustainability or settlement factor (Factor A).

Towns name	Disagree (%)	Neutral (%)	Agree (%)
Chengguan	8	6	86
Haocheng	7	8	85
Hugo	7	10	83
Liancheng	7	12	81
Liuji	8	14	78
Renqiao	8	13	79
Wangzhuang	6	12	82
Xinmaqiao	4	14	82

**Table 5:** Contingency values of health-related living environment (Factor B).

Towns name	Disagree (%)	Neutral (%)	Agree (%)
Chengguan	1	3	96
Haocheng	0	2	98
Hugo	0	2	98
Liancheng	0	0	100
Liuji	0	2	98
Renqiao	0	1	99
Wangzhuang	0	5	95
Xinmaqiao	1	1	98

**Table 6:** Expected frequencies.

**Expected frequencies**			
	**Disagree**	**Neutral**	**Agree**

*Factor A (human settlement)*			
Chengguan	6.72	10.8	82.48
Haocheng	6.92	11.12	85.96
Hugo	6.36	10.24	78.4
Liancheng	6.36	10.24	78.4
Liuji	6.8	10.96	84.24
Renqiao	6.96	11.2	85.84
Wangzhuang	6.76	10.88	83.36
Xinmaqiao	6.4	10.32	79.28

*Factor B (heath-related living environment)*			
Chengguan	1.68	2.72	96.6
Haocheng	1.72	2.78	98.5
Hugo	1.6	2.58	95.82
Liancheng	1.6	2.58	95.82
Liuji	1.7	2.74	100.56
Renqiao	1.74	2.8	100.46
Wangzhuang	1.68	2.72	96.6
Xinmaqiao	1.6	2.58	95.82

Chi-square	227.52		
Degree of freedom	4		
*P* value	0.071		

## Data Availability

All data are available within the article.

## References

[1] Ma D., Chen X., Wei H., Song Y. (2023). *Promoting the Development of Urban–Rural Integration BT - Rural Revitalization in China: A Socialist Road with Chinese Characteristics*.

[2] Chen M., Zhou Y., Huang X., Ye C. (2021). The Integration of New-type Urbanization and Rural Revitalization Strategies in China: Origin, Reality and Future Trends. *Land*.

[3] Shi J., Yang X. (2022). Sustainable Development Levels and Influence Factors in Rural China Based on Rural Revitalization Strategy. *Sustainability*.

[4] Mei Y., Miao J., Lu Y. (2022). Digital Villages Construction Accelerates High-Quality Economic Development in Rural China through Promoting Digital Entrepreneurship. *Sustainability*.

[5] Jie F. (2020). High-quality Development of National Territory Space Governance and Regional Economic Layout during 14th Five-Year Plan in China, Bull. Chinese Acad. *Scientia*.

[6] Gao J. (2022). Cultural Industry Development from Entrepreneurship under the Background of Rural Revitalization Strategy. *Frontiers in Psychology*.

[7] Ye L., Wu Z., Wang T., Ding K., Chen Y. (2022). Villagers’ Satisfaction Evaluation System of Rural Human Settlement Construction: Empirical Study of Suzhou in China’s Rapid Urbanization Area. *International Journal of Environmental Research and Public Health*.

[8] Chen J. (2007). Rapid Urbanization in China: A Real Challenge to Soil Protection and Food Security. *Catena*.

[9] Liu Y., Liu Y., Chen Y., Long H. (2010). The Process and Driving Forces of Rural Hollowing in China under Rapid Urbanization. *Journal of Geographical Sciences*.

[10] Tang W., Pei Y., Zheng H., Zhao Y., Shu L., Zhang H. (2022). Twenty Years of China’s Water Pollution Control: Experiences and Challenges. *Chemosphere*.

[11] Vennemo H., Aunan K., Lindhjem H., Seip H. M. (2009). Environmental Pollution in China: Status and Trends. *Review of Environmental Economics and Policy*.

[12] Liang W., Yang M. (2019). Urbanization, Economic Growth and Environmental Pollution: Evidence from China. *Sustainable Computing: Informatics and Systems*.

[13] Zhang K., Wen Z. (2008). Review and Challenges of Policies of Environmental Protection and Sustainable Development in China. *Journal of Environmental Management*.

[14] Mumtaz S., Rana J. N., Choi E. H., Han I. (2022). Microwave Radiation and the Brain: Mechanisms, Current Status, and Future Prospects. *International Journal of Molecular Sciences*.

[15] Rana J. N., Mumtaz S., Choi E. H., Han I. (2023). ROS Production in Response to High-Power Microwave Pulses Induces P53 Activation and DNA Damage in Brain Cells: Radiosensitivity and Biological Dosimetry Evaluation. *Frontiers in Cell and Developmental Biology*.

[16] Rana J. N., Mumtaz S., Han I., Choi E. H. (2024). Formation of Reactive Species via High Power Microwave Induced DNA Damage and Promoted Intrinsic Pathway-Mediated Apoptosis in Lung Cancer Cells: An In Vitro Investigation. *Fundamental Research*.

[17] Cheng Z., Li L., Liu J. (2019). The Effect of Information Technology on Environmental Pollution in China. *Environmental Science & Pollution Research*.

[18] Ma L., Liu S., Fang F., Che X., Chen M. (2020). Evaluation of Urban-Rural Difference and Integration Based on Quality of Life. *Sustainable Cities and Society*.

[19] Zhou Y., Li Y., Xu C. (2020). Land Consolidation and Rural Revitalization in China: Mechanisms and Paths. *Land Use Policy*.

[20] Kalantaridis C., Bika Z. (2006). In-migrant Entrepreneurship in Rural England: beyond Local Embeddedness. *Entrepreneurship & Regional Development*.

[21] Zhan Z., Cenci J., Zhang J. (2022). Frontier of Rural Revitalization in China: A Spatial Analysis of National Rural Tourist Towns. *Land*.

[22] Chengjun S., Renhua S., Zuliang S. (2022). Construction Process and Development Trend of Ecological Agriculture in China. *Acta Ecologica Sinica*.

[23] Zhang T., Duan Y., Jiao Z. (2022). Towards Improving Rural Living Environment for Chinese Cold Region Based on Investigation of Thermal Environment and Space Usage Status. *Buildings*.

[24] Rana J. N., Mumtaz S., Han I., Choi E. H. (2024). Harnessing the Synergy of Nanosecond High-Power Microwave Pulses and Cisplatin to Increase the Induction of Apoptosis in Cancer Cells through the Activation of ATR/ATM and Intrinsic Pathways. *Free Radical Biology and Medicine*.

[25] Alotaibi B. S., Elnaklah R., Agboola O. P. (2024). Enhancing Najran’s Sustainable Smart City Development in the Face of Urbanization Challenges in Saudi- Arabia. *Journal of Asian Architecture and Building Engineering*.

[26] Agboola O. P., Alotaibi B. S., Dodo Y. A., Abuhussain M. A., Abuhussain M. (2024). Built Environment Transformation in Nigeria: the Effects of a Regenerative Framework. *Journal of Asian Architecture and Building Engineering*.

[27] Agboola O. P., Tunay M. (2023). Urban Resilience in the Digital Age: The Influence of Information-Communication Technology for Sustainability. *Journal of Cleaner Production*.

[28] Agboola O. P., Azizul M. F., Rasidi M. H., Said I. (2018). The Cultural Sustainability of Traditional Market Place in Africa: A New Research Agenda. *Journal of Rural Studies*.

[29] Agboola O. P., Rasidi M. H., Said I., Abogan S. O., Adejuwon A. S. (2018). Morphological and GIS-Based Land Use Analysis: A Critical Exploration of a Rural Neighborhood. *Journal of Contemporary Urban Affairs*.

[30] Agboola O. P., Rasidi M. H., Said I. (2017). Adolescents’ Sense of Community and Involvement in Playground Activities: Panacea to Ameliorate Social Vices and Delinquencies. *International Journal of Built Environment and Sustainability*.

[31] Agboola O. P. (2022). The Significance of Rural Markets as a Public Space in Nigeria. *Habitat International*.

[32] Hafeez A., Dangel W. J., Ostroff S. M. (2023). The State of Health in Pakistan and Its Provinces and Territories, 1990–2019: A Systematic Analysis for the Global Burden of Disease Study 2019. *Lancet Global Health*.

[33] Local Burden of Disease Hiv Collaborators, Collaborators D. H. I. V. (2021). Mapping Subnational HIV Mortality in Six Latin American Countries with Incomplete Vital Registration Systems. *BMC Medicine*.

[34] Farzadfar F., Naghavi M., Sepanlou S. G. (2022). Health System Performance in Iran: a Systematic Analysis for the Global Burden of Disease Study 2019. *The Lancet*.

[35] Meng Q., Yan Z., Abbas J., Shankar A., Subramanian M. (2023). Human–Computer Interaction and Digital Literacy Promote Educational Learning in Pre-school Children: Mediating Role of Psychological Resilience for Kids’ Mental Well-Being and School Readiness. *International Journal of Human-Computer Interaction*.

[36] Abbas J., Mubeen R., Iorember P. T., Raza S., Mamirkulova G. (2021). Exploring the Impact of COVID-19 on Tourism: Transformational Potential and Implications for a Sustainable Recovery of the Travel and Leisure Industry. *Current Research in Behavioral Sciences*.

[37] Abbas J., Wang D., Su Z., Ziapour A. (2021). The Role of Social Media in the Advent of COVID-19 Pandemic: Crisis Management, Mental Health Challenges and Implications. *Risk Management and Healthcare Policy*.

[38] Schumacher A.E., Kyu H. H., Aali A. (2024). Global Age-sex-specific Mortality, Life Expectancy, and Population Estimates in 204 Countries and Territories and 811 Subnational Locations, 1950–2021, and the Impact of the COVID-19 Pandemic: A Comprehensive Demographic Analysis for the Global Bur. *Lancet*.

[39] Mumtaz S., Javed R., Rana J. N., Iqbal M., Choi E. H. (2024). Pulsed High Power Microwave Seeds Priming Modulates Germination, Growth, Redox Homeostasis, and Hormonal Shifts in Barley for Improved Seedling Growth: Unleashing the Molecular Dynamics. *Free Radical Biology and Medicine*.

[40] Xianwang Tan Jaffar Abbas K. A.-S. L. P., Shah S. A. R. (2024). The Role of Digital Management and Smart Technologies for Sports Education in a Dynamic Environment: Employment, Green Growth, and Tourism. *Journal of Urban Technology*.

[41] Shuja J. (2022). Kanwar Hamza and Abbas, Criminal Recidivism in Pakistan: A Grounded Theory of Social & Environmental Causes and Psychological Consequences, Nature-Nurture J. *The Psychologist*.

[42] Abbas J., Balsalobre-Lorente D., Amjid M. A., Al-Sulaiti K., Al-Sulaiti I., Aldereai O. (2024). Financial Innovation and Digitalization Promote Business Growth: The Interplay of Green Technology Innovation, Product Market Competition and Firm Performance. *Innovation and Green Development*.

[43] Abbas J., Mamirkulova G., Al-Sulaiti I., Al-Sulaiti K. I., Dar I. B. (2024). Mega-infrastructure Development, Tourism Sustainability and Quality of Life Assessment at World Heritage Sites: Catering to COVID-19 Challenges. *Kybernetes*.

[44] Chen J., Chen S. (2015). Mental Health Effects of Perceived Living Environment and Neighborhood Safety in Urbanizing China. *Habitat International*.

[45] Wang L., Yang Y., Zhu J. (2019). Professional Identity and Mental Health of Rural-Oriented Tuition-Waived Medical Students in Anhui Province, China. *BMC Medical Education*.

[46] Mketo A. R., Ringo C. J., Nuhu S., Mpambije C. J. (2022). Enhancing Community Participation for Environmental Health Improvement in Rural Tanzania: Evidence from Bukombe District. *Evaluation and Program Planning*.

[47] Pan D., Yu Y., Ji K. (2024). The Impact of Rural Living Environment Improvement Programs on the Subjective Well-Being of Rural Residents in China. *Humanities and Social Sciences Communications*.

[48] Manzini E. (2015). *Design, when Everybody Designs: An Introduction to Design for Social Innovation*.

[49] Jiao Y., Cao P. (2023). Research on Optimization of Project Design Management Process Based on BIM. *Buildings*.

[50] Chen J., Zeng H., Gao Q. (2023). Using the Sustainable Development Capacity of Key Counties to Guide Rural Revitalization in China. *International Journal of Environmental Research and Public Health*.

[51] Zhang J., Mauzerall D. L., Zhu T., Liang S., Ezzati M., Remais J. V. (2010). Environmental Health in China: Progress towards Clean Air and Safe Water. *The Lancet*.

[52] Wang P., Qin X., Li Y. (2021). Satisfaction Evaluation of Rural Human Settlements in Northwest China: Method and Application. *Land*.

[53] Gao B., Hu Z. (2022). What Affects the Level of Rural Human Settlement? A Case Study of Tibet, China. *Sustainability*.

[54] Xu Y., Zhang R., Wu W. (2023). Dynamic Changes and Driving Factors of Rural Settlements at the County Level in a Rapidly Urbanizing Province of China from 2000 to 2020. *Frontiers of Environmental Science*.

[55] Hu X., Cook S., Salazar M. A. (2008). Internal Migration and Health in China. *The Lancet*.

[56] Zhang Y., Zhang J., Ye Y., Wu Q., Jin L., Zhang H. (2016). Residents’ Environmental Conservation Behaviors at Tourist Sites: Broadening the Norm Activation Framework by Adopting Environment Attachment. *Sustainability*.

[57] Wei C., Meng J., Zhu L., Han Z. (2023). Assessing Progress towards Sustainable Development Goals for Chinese Urban Land Use: A New Cloud Model Approach. *Journal of Environmental Management*.

[58] Liu J., Zheng B., Tang H. (2023). The Science of Rural Human Settlements: a Comprehensive Overview. *Frontiers of Environmental Science*.

[59] Bulakh I. (2022). Sustainable Development in the Context of the Architecture of Environmental Friendly Medical Centers in Rural Areas (Case for Ukraine). *IOP Conference Series: Earth and Environmental Science*.

[60] Wang B., Gu K., Dong D., Fang Y., Tang L. (2022). Analysis of Spatial Distribution of CVD and Multiple Environmental Factors in Urban Residents. *Computational Intelligence and Neuroscience*.

[61] Gu K., Li Y., Jia X., Liu C. (2023). Multiple Impacts of Urban Built and Natural Environment on Lung Cancer Incidence: A Case Study in Bengbu. *Journal of Healthcare Engineering*.

[62] Tang J., Gu K., Mi J. (2022). Spatio-temporal Distribution Characteristics and Environmental Impact Factors of Lung Cancer Mortality: A Case Study of Yuhui District in Bengbu City, China. *Chinese Geographical Science*.

[63] Xiao M., Bao F., Wang S., Cui F. (2016). Water Quality Assessment of the Huaihe River Segment of Bengbu (China) Using Multivariate Statistical Techniques. *Water Resources Series*.

[64] Shen X., Chen B., Leibrecht M., Du H. (2022). The Moderating Effect of Perceived Policy Effectiveness in Residents’ Waste Classification Intentions: A Study of Bengbu, China. *Sustainability*.

[65] Hamzehnejadi Y., Mangolian Shahrbabaki P., Alnaiem M. (2024). The Impact of Massage and Dry Cupping on Dysrhythmia in Cardiac Patients: A Randomized Parallel Controlled Trial. *Journal of Bodywork and Movement Therapies*.

[66] Micah A. E., Bhangdia K., Cogswell I. E. (2023). Global Investments in Pandemic Preparedness and COVID-19: Development Assistance and Domestic Spending on Health between 1990 and 2026. *Lancet Global Health*.

[67] Yadav R., Zaman K., Mishra A. (2022). Health Seeking Behavior and Healthcare Utilization in a Rural Cohort of North India. *Healthcare*.

[68] Quilliam C., Glenister K., Ervin K., Weller-Newton J. (2023). Revisiting Rural Healthcare Access Through Held’s Ethics of Care. *Social Theory & Health*.

[69] Baker E. L., Potter M. A., Jones D. L. (2005). THE PUBLIC HEALTH INFRASTRUCTURE AND OUR NATION’S HEALTH. *Annual Review of Public Health*.

[70] Ekonomou E., Fan L., Buchanan W., Thuemmler C. An Integrated Cloud-Based Healthcare Infrastructure.

[71] Tumrate C. S., Saini D. K., Gupta P., Mishra D. (2023). Evolutionary Computation Modelling for Structural Health Monitoring of Critical Infrastructure. *Archives of Computational Methods in Engineering*.

[72] Mbunge E., Batani J., Gaobotse G., Muchemwa B. (2022). Virtual Healthcare Services and Digital Health Technologies Deployed during Coronavirus Disease 2019 (COVID-19) Pandemic in South Africa: a Systematic Review. *Global Health Journal*.

[73] Smith J. A., Canuto K., Canuto K. (2022). Advancing Health Promotion in Rural and Remote Australia: Strategies for Change. *Health Promotion Journal of Australia*.

[74] Lee E. K., Chen C.-H., Pietz F., Benecke B. (2009). Modeling and Optimizing the Public-Health Infrastructure for Emergency Response. *Interfaces*.

[75] Carroll C., Planey A., Kozhimannil K. B. (2022). Reimagining and Reinvesting in Rural Hospital Markets. *Health Services Research*.

[76] Tang J., Ruan H., Wang C., Xu W., Li C., Dong X. (2022). Social Network, Cognition and Participation in Rural Health Governance. *International Journal of Environmental Research and Public Health*.

[77] Zhang J., Chandola T., Zhang N. (2022). Understanding the Longitudinal Dynamics of Rural–Urban Mental Health Disparities in Later Life in China. *Aging & Mental Health*.

[78] Jakovljevic M., Pallegedara A., Vinayagathasan T., Kumara A. S. (2022). Editorial: Inequality in Healthcare Utilization and Household Spending in Developing Countries. *Frontiers in Public Health*.

[79] Zuo T., Zhang F., Zhang J., Gao L., Yu S. (2022). Rocky Desertification Poverty in Southwest China: Progress, Challenges and Enlightenment to Rural Revitalization. *Journal of Geographical Sciences*.

[80] Liu Y. (2018). Introduction to Land Use and Rural Sustainability in China. *Land Use Policy*.

[81] Monfreda C., Wackernagel M., Deumling D. (2004). Establishing National Natural Capital Accounts Based on Detailed Ecological Footprint and Biological Capacity Assessments. *Land Use Policy*.

[82] Wang D., Shen Y. (2022). Sanitation and Work Time: Evidence from the Toilet Revolution in Rural China. *World Development*.

[83] Wang S., Guo Y., He Z. (2023). Analysis on the Measurement and Dynamic Evolution of Multidimensional Return to Poverty in Chinese Rural Households. *Social Indicators Research*.

[84] Li D., Xu F., Chen Z., Xie X., Fan K., Zeng Z. (2024). Fine Simulation of PM2.5 Combined with NPP-VIIRS Night Light Remote Sensing and Mobile Monitoring Data. *Science of the Total Environment*.

[85] Tan X., Liu X., Shao H. (2017). Healthy China 2030: A Vision for Health Care. *Value in Health Regional Issues*.

[86] Zhang X., Abbas J., Shahzad M. F., Shankar A., Ercisli S., Dobhal D. C. (2024). Association between Social Media Use and Students’ Academic Performance through Family Bonding and Collective Learning: The Moderating Role of Mental Well-Being. *Education and Information Technologies*.

[87] Shoib S., Gaitán Buitrago J. E. T., Shuja K. H. (2022). Suicidal Behavior Sociocultural Factors in Developing Countries during COVID-19. *L'Encéphale*.

[88] Steinmetz J. D., Seeher K. M., Schiess N. (2024). Global, Regional, and National Burden of Disorders Affecting the Nervous System, 1990–2021: a Systematic Analysis for the Global Burden of Disease Study 2021. *The Lancet Neurology*.

